# Front-line treatment of multiple myeloma

**DOI:** 10.1097/HS9.0000000000000242

**Published:** 2019-06-30

**Authors:** Michele Cavo, Paola Tacchetti, Elena Zamagni

**Affiliations:** Seràgnoli Institute of Hematology, *Alma Mater Studiorum*-Bologna University School of Medicine, S.Orsola-Malpighi Hospital, Bologna, Italy

**Take home messages**

- Incorporation of novel agents into the treatment paradigms for fit patients with newly diagnosed multiple myeloma has led to a remarkable increase in the rates of complete response and minimal residual disease negative status, ultimately translating into near tripling of median overall survival of this disease.
- In frail individuals who cannot tolerate multi-drug combinations treatment goals include improvement in the quality of life and avoidance of early treatment discontinuation due to drug toxicities.

## Introduction

In a patient with newly diagnosed (ND) multiple myeloma (MM), start of therapy is immediately required if any one or more of myeloma defining events are detected.^1^ The subsequent step in the selection of the most appropriate treatment strategy is to define if the patient is able, or not, to tolerate high-dose therapy (HDT) with autologous stem-cell transplantation (ASCT). Eligibility for ASCT is usually based on chronological age, performance status and comorbidities. In ASCT-ineligible patients, unfitness and frailty must be adequately assessed before making treatment decision. Geriatric assessment metrics, although underemployed in routine care, are a valuable tool to identify frailty and vulnerability.^2^ In fit patients who can tolerate novel agent-based therapies at full doses, followed or not by ASCT, key treatment end points are to achieve undetectable minimal residual disease (MRD) and to sustain MRD negative status, as a way to significantly enhance progression-free survival (PFS) and overall survival (OS).^3^

## Current state of the art: ASCT candidates

Induction therapy in preparation for ASCT should include the first-in-class proteasome inhibitor (PI) bortezomib combined with dexamethasone (VD) and a third drug.^4^ This latter should be preferably an immunomodulatory agent (IMiD), like thalidomide (VTD) or lenalidomide (VRD), but cyclophosphamide (VCD) or doxorubicin (PAD) might be alternative options^5▪^^,^^6^ (Table ^1^). Several studies are currently investigating the efficacy and toxicity of 3 or 4 drug regimens incorporating a second generation PI like carfilzomib plus an IMiD and dexamethasone, or the first-in-class anti-CD38 monoclonal antibody (mAb) daratumumab combined with VTD or VRD. The number of preplanned cycles to be given before peripheral blood SC harvest is usually four. However, an increase up to 6 cycles may be considered, provided that there is no signal of emerging treatment-related peripheral neuropathy and response continues to deepen. Although the role of upfront ASCT has been debated in the current era,^7^ it still remains the standard of care for eligible patients in European countries, based on the results from several prospective studies showing a significantly longer PFS with HDT in comparison with standard-dose therapies incorporating bortezomib.^8▪^^,^^9^ The lack of OS benefit with upfront ASCT in studies not yet mature for OS analysis at the time of their initial publication does not justify the choice to delay ASCT at the time of relapse, since a possible divergence between survival curves might emerge with longer follow up. The role of double, or tandem, ASCT remains controversial, as confirmed by opposite results from 2 randomized studies recently conducted in Europe and the United States (US), partly related to major differences in their design.^10^,^11^ A preplanned interim analysis of the European Myeloma Network (EMN) 02 study, showed that double ASCT following short-term bortezomib-based induction therapy (a design closely reflecting the current practice in most European centers) prolonged PFS and OS in comparison with a single ASCT.^9^ Differences in outcomes between the 2 treatment arms were particularly remarkable in patients at high-risk according to adverse cytogenetics or advanced International Staging System (ISS) disease stage. Short-term consolidation therapy after ASCT more frequently comprises the same drug or multi-agent combination previously administered as induction before HDT and is aimed at further enhancing the rate and depth of response, as well as the length of PFS. Improved clinical outcomes reported with consolidation vs no consolidation therapy in the EMN02 study are currently reflected in the clinical practice across several European groups.^12^ Whether prolonged induction therapy may abrogate the potential benefits seen with post-ASCT consolidation treatment following a limited number of induction cycles, as typically planned in European countries, remains an area of debate.^7^ Maintenance therapy after ASCT is usually given until progression or for up to 2 to 3 years, in order to sustain the duration of response or MRD negative status and to prolong OS, while keeping toxicity minimal. Lenalidomide is actually the only novel agent approved by the European Medicines Agency (EMA) as maintenance therapy following ASCT, on the basis of the results of a meta-analysis of 3 randomized clinical trials designed to compare lenalidomide (R) vs placebo or observation.^13▪^ PFS and OS benefits seen with R maintenance in the intention-to-treat population were retained across different subgroups of patients, although those with ISS stage 3 and a high-risk cytogenetic profile were likely to fail a significant improvement in OS. By the opposite, in several studies maintenance with bortezomib alone for up to 2 years following a VD-based induction therapy or with bortezomib and R improved or overcame the poor prognosis imparted by del(17p) or t(4;14), suggesting that exposure to PI after ASCT might be of benefit in patients carrying high-risk cytogenetics.^14^,^15^ Unfortunately, the lack of phase III studies designed to prospectively address this issue or, more generally, to compare bortezomib maintenance vs placebo or observation in NDMM patients who have undergo ASCT prevents any formal recommendation about V use in this setting. More recently, the second generation oral PI ixazomib has been reported to prolong PFS in comparison with placebo following ASCT. These data taken in aggregate confirm the role of upfront ASCT in NDMM patients and provide evidence supporting the remarkable advances seen with incorporation of novel agents into HDT, ultimately leading to OS estimates at 10 years in the 60% range (Table ^1^).

**Table 1:**
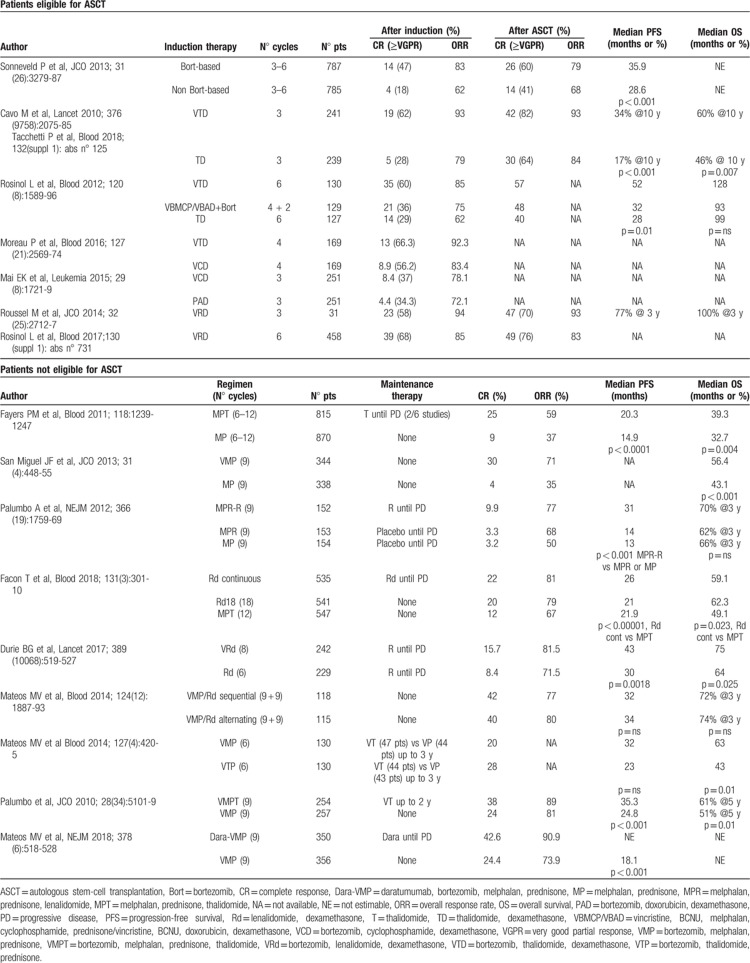
Design of clinical trials and reported outcomes for patients with newly diagnosed multiple myeloma who are eligible or not eligible for autologous stem-cell transplantation.

## Current state of the art: non-ASCT candidates

Basically, treatment options for ASCT-ineligible patients can either include or exclude alkylators. Treatment approaches can also differ in terms of exposure duration to therapeutic agents, which can be either fixed or continuous until progression or toxicity. Over the past years, melphalan-prednisone (MP) was combined with thalidomide (MPT) or bortezomib (VMP) or R (MPR) in several phase III studies^16▪^,^17^,^18^,^19^ (Table ^1^). All these triplets showed superior efficacy in comparison with MP and were approved by EMA for the treatment of patients who are not candidates for ASCT. Due to the high rate of severe neutropenia observed during induction therapy with MPR and the lack of benefit with this regimen in patients older than 75 years of age, its routine use has been limited. R-dexamethasone (Rd) until progression (Rd continuous) is another recent EMA-approved therapeutic option in the non-ASCT setting^20▪^ (Table ^1^). VMP and Rd represent at this time the most popular gold standard therapies offered to ASCT-ineligible NDMM patients in European countries. The choice of one regimen vs another is based on various factors related to the disease itself (for example, renal failure and adverse cytogenetics which favor VMP) or to the host, including patient's fitness or frailty, as well as preference of the single individual. According to the latest guidelines provided by the European Society for Medical Oncology, alternative treatment options in the non-ASCT setting include VCD, cyclophosphamide-thalidomide-dexamethasone (CTD) and bendamustine-prednisone (BP).^6^ Notably, not all these treatments have been backed by phase III studies and are EMA-approved, while some of them have got approval in particular clinical conditions (for example, BP in patients with peripheral neuropathy precluding their exposure to thalidomide or bortezomib). More recently, addition of bortezomib to Rd induction followed by Rd continuous (VRd) significantly improved outcomes in comparison with Rd continuous, and is likely to become a new standard of care, once approved in Europe^21^ (Table ^1^). By the opposite, alternative treatment approaches including the sequential or alternating administration of both VMP and Rd for a fixed number of cycles, as well as continuous exposure to V-prednisone or V-thalidomide following induction therapy with either VMP or VMP plus thalidomide did not got approval by EMA and, although of clinical benefit, are not commonly used in European centers^17^ (Table ^1^). Nevertheless, a paradigm shift toward the use of continuous therapies in the next few years can be predicted, in light of recent phase III studies that have established new gold standard treatments. In two of these studies, addition of daratumumab either to VMP induction followed by daratumumab until progression or to Rd continuous significantly improved clinical outcomes in comparison with VMP and Rd, respectively^18^ (Table ^1^). In addition, both Rd and VRd have represented the backbone of other alkylator-free triplets or quadruplets which have been, or are currently being, explored in combination with ixazomib or daratumumab or the monoclonal antibodies elotuzumab and isatuximab targeting SLAMF7 and CD38 antigens, respectively. In summary, incorporation of novel agents at full doses into the treatment paradigms for fit ASCT-ineligible patients with NDMM has led to unprecedented rates of complete response and MRD negativity, ultimately translating into extended PFS and OS. Treatment goals for frail individuals who cannot tolerate multi-drug combinations at full doses are shifted towards patient-centered outcomes, and include improvement in the quality of life and avoidance of early treatment discontinuation due to drug toxicities.

## Future perspectives

The treatment landscape for NDMM has been transformed with the introduction of PIs, IMiDs and mAbs, and still continues to evolve. Novel agents combined with each other and/or with standard of care regimens are already in the clinic for both ASCT-eligible and -ineligible patients. In the meantime, other newer therapies are under active evaluation. Treatments incorporating novel drugs with different, and often complementary, mechanisms of action bring an increased anti-MM activity with lower off target toxicity, and hold promise to further enhance the probability and depth of response. Achievement of MRD negativity rates, actually in the 30% to 60% range in ASCT-ineligible and -eligible patients, respectively, will hopefully translate in the coming years into furtherly extended PFS and OS, and are likely to offer a chance of cure to a fraction of NDMM patients at standard risk.
